# Structure of the Pf12 and Pf41 heterodimeric complex of *Plasmodium falciparum* 6-cysteine proteins

**DOI:** 10.1093/femsmc/xtac005

**Published:** 2022-02-16

**Authors:** Melanie H Dietrich, Li-Jin Chan, Amy Adair, Coralie Boulet, Matthew T O'Neill, Li Lynn Tan, Sravya Keremane, Yee-Foong Mok, Alvin W Lo, Paul Gilson, Wai-Hong Tham

**Affiliations:** Infectious Diseases and Immune Defence Division, The Walter and Eliza Hall Institute of Medical Research, Parkville, Victoria3052, Australia; Department of Medical Biology, The University of Melbourne, Melbourne, Victoria, 3052, Australia; Infectious Diseases and Immune Defence Division, The Walter and Eliza Hall Institute of Medical Research, Parkville, Victoria3052, Australia; Department of Medical Biology, The University of Melbourne, Melbourne, Victoria, 3052, Australia; Infectious Diseases and Immune Defence Division, The Walter and Eliza Hall Institute of Medical Research, Parkville, Victoria3052, Australia; Malaria Virulence and Drug Discovery Group, Burnet Institute, Melbourne, 3004, Australia; Infectious Diseases and Immune Defence Division, The Walter and Eliza Hall Institute of Medical Research, Parkville, Victoria3052, Australia; Infectious Diseases and Immune Defence Division, The Walter and Eliza Hall Institute of Medical Research, Parkville, Victoria3052, Australia; Infectious Diseases and Immune Defence Division, The Walter and Eliza Hall Institute of Medical Research, Parkville, Victoria3052, Australia; Department of Biochemistry and Molecular Biology and Bio21 Molecular Science and Biotechnology Institute, The University of Melbourne, Melbourne, 3052, Australia; Infectious Diseases and Immune Defence Division, The Walter and Eliza Hall Institute of Medical Research, Parkville, Victoria3052, Australia; Malaria Virulence and Drug Discovery Group, Burnet Institute, Melbourne, 3004, Australia; Infectious Diseases and Immune Defence Division, The Walter and Eliza Hall Institute of Medical Research, Parkville, Victoria3052, Australia; Department of Medical Biology, The University of Melbourne, Melbourne, Victoria, 3052, Australia

**Keywords:** malaria, X-ray crystallography, Nanobodies, 6-cysteine proteins, *Plasmodium falciparum*, blood stages

## Abstract

During the different stages of the *Plasmodium* life cycle, surface-associated proteins establish key interactions with the host and play critical roles in parasite survival. The 6-cysteine (6-cys) protein family is one of the most abundant surface antigens and expressed throughout the *Plasmodium falciparum* life cycle. This protein family is conserved across *Plasmodium* species and plays critical roles in parasite transmission, evasion of the host immune response and host cell invasion. Several 6-cys proteins are present on the parasite surface as hetero-complexes but it is not known how two 6-cys proteins interact together. Here, we present a crystal structure of Pf12 bound to Pf41 at 2.85 Å resolution, two *P. falciparum* proteins usually found on the parasite surface of late schizonts and merozoites. Our structure revealed two critical interfaces required for complex formation with important implications on how different 6-cysteine proteins may interact with each other. Using structure-function analyses, we identified important residues for Pf12-Pf41 complex formation. In addition, we generated 16 nanobodies against Pf12 and Pf41 and showed that several Pf12-specific nanobodies inhibit Pf12-Pf41 complex formation. Using X-ray crystallography, we were able to describe the structural mechanism of an inhibitory nanobody in blocking Pf12-Pf41 complex formation. Future studies using these inhibitory nanobodies will be useful to determine the functional role of these two 6-cys proteins in malaria parasites.

## Teaser

Malaria is a deadly disease that causes over 400 000 deaths worldwide. During its life cycle, malaria parasites exist in several forms across its different life stages and present diverse surface coat proteins as antigenic targets. One of the more abundant surface coat proteins of *Plasmodium falciparum*, the most lethal malaria parasite species, is the 6-cysteine protein family. Several members of this family are implicated in critical functions in fertilization, transmission, immune evasion and host cell invasion. Here, using structural biology, we provide an understanding on how two 6-cysteine proteins, Pf12 and Pf41 which are abundant blood stage merozoite proteins, bind to each other using interfaces between their respective 6-cysteine domains and is further anchored by an insertion of a Pf41 loop into the second 6-cysteine domain of Pf12. We found that nanobodies against Pf12 were able to block complex formation and structural analyses showed that a nanobody loop binds to a region, which normally binds the Pf41 insertion loop, thus resulting in steric hindrance in one of the critical interaction sites. Our study provides new structural insights on the 6-cysteine proteins that will be important to progress our understanding on how they function during the malaria parasite life cycle.

## Introduction

During the different extracellular stages of the *Plasmodium* life cycle, the malaria parasite expresses surface coat proteins, which establish key interactions with host proteins and play important roles in parasite survival. The members of the 6-cysteine (6-cys) protein family represent some of the more abundant surface proteins and have crucial roles in host-cell entry, parasite sexual development and immune evasion strategies (van Dijk et al. 2010, Kennedy et al. 2016, Manzoni et al. 2017, Arredondo et al. 2018, Molina-Cruz et al. 2020). In *Plasmodium falciparum*, the most virulent malaria parasite in humans, the 6-cys family has 14 members, Pfs230, Pfs48/45, Pf230p, PfP47, PfSOP12, Pf52, Pf36, PfLISP2, PfB9, Pf12, P12p, Pf41, Pf38, and Pf92, that are conserved across *Plasmodium* species and expressed throughout the life cycle in a stage-specific manner (Aurrecoechea et al. 2009). Most family members are localised on the parasite surface with some tethered to the membrane via glycosylphosphatidylinositol (GPI)-anchors (Sanders et al. 2005, Gilson et al. 2006). The 6-cys proteins contain between 1 and 14 6-cys domains, which are often present as tandem pairs (Gerloff et al. 2005). The 6-cys domain consists of a β-sandwich fold formed by two β-sheets of mixed parallel and anti-parallel β-strands, with up to six positionally conserved cysteines that form disulfide bonds (Carter et al. 1995, Gerloff et al. 2005, Arredondo et al. 2012). The 6-cys domain is found in proteins of all members of the Aconoidasidan clade of Apicomplexa and is evolutionary related to the SAG1-related sequence (SRS) domain of *Toxoplasma gondii*. SRS domains are present in GPI-anchored surface proteins with functions in cell-attachment and immune evasion (He et al. 2002, Jung et al. 2004, Crawford et al. 2009, Arredondo et al. 2012).

In *P. falciparum*, members of the 6-cys family are associated with diverse and critical roles of the *Plasmodium* life cycle. LISP2 and B9 are liver-stage proteins and important for parasite development within hepatocytes (Orito et al. 2013, Annoura et al. 2014, van Schaijk et al. 2014). Pfs47 is on the surface of female gametocytes, zygotes, and ookinetes and involved in evasion of the mosquito immune system (Molina-Cruz et al. 2020). Pf92 is localised on blood-stage merozoites and recruits human complement regulator Factor H to circumvent the human host immune system (Kennedy et al. 2016). Pfs230 and Pfs48/45 are leading transmission blocking vaccine candidates expressed on the surface of male and female gametes. Pfs48/45 is GPI-anchored and forms a stable complex with secreted Pfs230 (Kumar 1987, Kumar and Wizel 1992). They are crucial for male fertility and antibodies against them are effective in blocking transmission between human and mosquito (Quakyi et al. 1987, van Dijk et al. 2001, van Dijk et al. 2010, Theisen et al. 2017). Pf36 and Pf52 are expressed in the sporozoite stages and essential for productive invasion of hepatocytes (Ishino et al. 2005). Both are secreted but only Pf52 is predicted to be GPI-anchored. P36 has been implicated to mediate interactions with a range of hepatocyte receptors in a *Plasmodium* species dependent manner and partially co-localised and co-immunoprecipitated with P52 (Kaushansky et al. 2015, Manzoni et al. 2017, Arredondo et al. 2018). Genetically modified parasites lacking *p36* and *p52* are currently under investigation as potential live attenuated vaccine (Kublin et al. 2017).

On the merozoite surface, Pf12 is one of the most abundant GPI-anchored proteins and forms a heterodimeric complex with the secreted 6-cys protein Pf41 (Gilson et al. 2006, Taechalertpaisarn et al. 2012, Tonkin et al. 2013). Both Pf12 and Pf41 are strongly recognised by immune sera of naturally infected individuals (Elliott et al. 1990, Sanders et al. 2005, Tetteh et al. 2009, Richards et al. 2013, Tonkin et al. 2013). However, Pf12 and Pf41 knockout parasites have no growth defects during the blood stages and polyclonal antibodies against either Pf12 or Pf41 showed no growth inhibition (Taechalertpaisarn et al. 2012). Both Pf12 and Pf41 contain two 6-cys domains and the crystal structures of the individual proteins have been determined (Arredondo et al. 2012, Tonkin et al. 2013, Parker et al. 2015). The Pf41 structure alone revealed an intra-domain insertion called the inserted-domain-like region (ID) which when deleted abolished complex formation with Pf12 (Parker et al. 2015). While the structure of Pf12-Pf41 complex remains elusive, cross-linking studies predict an anti-parallel organisation of the two proteins (Tonkin et al. 2013, Parker et al. 2015).

Although several 6-cys proteins do form hetero-complexes, there have not been any structural analyses to understand how the respective 6-cys protein interact with each other. In this study, we determined the crystal structure of the Pf12-Pf41 heterodimer at 2.85 Å which highlighted critical interfaces required for complex formation. To identify critical residues important for Pf12-Pf41 assembly, we embarked on an extensive structural-function analysis. Furthermore, we characterised 16 nanobodies, which are single-domain antibodies isolated from immunised alpacas, for their specificity, affinity, ability to disrupt Pf12-Pf41 complex formation and in parasite growth assays. Using X-ray crystallography, we were able to describe the structural mechanism of an inhibitory nanobody in blocking Pf12-Pf41 complex formation.

## Results

### The crystal structure of the Pf12-Pf41 complex uncovers two distinct binding sites

**Figure 1.**
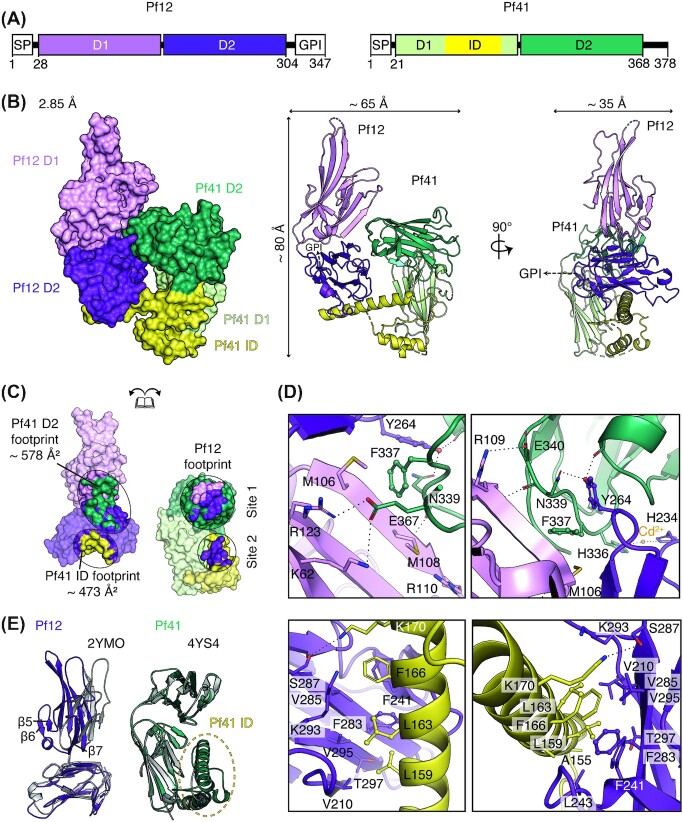
Crystal structure of the heterodimeric complex of Pf12-Pf41. **(A)** Schematic diagram of Pf12 and Pf41 (*left*, *right* respectively). SP, signal peptide; GPI, GPI-anchor; D1, N-terminal 6-cys domain; D2, C-terminal 6-cys domain; ID, inserted-domain like region. Residue numbers are indicated. **(B)** Structure of the Pf12-Pf41 complex in two orthogonal views as surface representation (*left*) and ribbon representation (*middle, right*). The two 6-cys domains, D1 and D2, are shown in light and dark purple for Pf12 and in light and dark green for Pf41 respectively. Within Pf41, the inserted domain-like region (ID) which is located between the last two β-strands of D1 is coloured yellow. A dashed arrow indicates the linker region of 17 residues between the C-terminus of Pf12 D1D2 and the predicted GPI-anchor attachment site (S321) which were not included in the crystallization construct. **(C)** The Pf12-Pf41interface. Pf12 residues that interact with the ID of Pf41 are shown in yellow; residues that interact with the C-terminal D2 domain of Pf41 are shown in dark green. Pf41 residues that are in contact with Pf12 D1 and D2 are shown in light and dark purple, respectively. Footprints for binding sites are defined by contacting residues within 5.0 Å. **(D)** Interactions between the Pf12 D1-D2 interdomain region and the Pf41 D2 domain in two different orientations (*top panels, left and right*). Interactions between the Pf12 D2 domain and the Pf41 ID shown in two different views (*bottom panels, left and right*). **(E**) Structural overlay of Pf12 D1D2 with the published unbound Pf12 crystal structure, PDB ID 2YMO, aligned based on the D2 domain (*left*). Structural overlay of Pf41 D1D2 with the published unbound Pf41 crystal structure, PDB ID 4YS4 (*right*).

To determine the crystal structure of the Pf12-Pf41 complex, we expressed a recombinant fragment of Pf12 encompassing residues 28–304 (Pf12 D1D2) and a recombinant fragment of Pf41 encompassing residues 21–368 (Pf41 D1D2) (Fig. 1A). Analytical ultracentrifugation experiments and size exclusion chromatography (SEC) show that they form a heterodimeric complex as previously described (Fig. S1, (Taechalertpaisarn et al. 2012, Parker et al. 2015)).

The Pf12-Pf41 crystal structure was determined at a resolution of 2.85 Å by molecular replacement and reveals a heterodimeric complex of approximately 80 × 65 × 35 Å in size (Fig. 1B and Table 1). The asymmetric unit contained one Pf12 D1D2 and one Pf41 D1D2 molecule. Both proteins fold into two 6-cys domains (D1 and D2) adopting a β-sandwich fold with three disulfide bonds per domain. Pf12 residues are resolved from N28-S304, except for a short loop within the D1 domain comprising residues 146–157. For Pf41, the structure starts from S20 and extends through to S368, with S20 being a residual residue of the fusion tag. The last two β-strands of the Pf41 D1 domain are connected via a 110 amino acid long insertion, termed inserted-domain-like region (ID), which spans amino acids 115 to 225. The Pf41 ID has two small (residues 116–129 and 196–200) and one long (residues 153–178) α-helices. Two segments, residues 132–150 and 192–228, remain unresolved as well as several side chains.

**Table 1.** Data collection and refinement statistics.

	Pf12-Pf41	Pf12–Nb G7
PDB ID	7S7Q	7S7R
**Data collection statistics**		
Wavelength (Å)	0.9536	0.9536
Resolution range (Å)	47.16–2.85 (3.02–2.85)	44.11–1.77 (1.88–1.77)
Space group	P4_3_2_1_2	P3_2_21
Cell axes (Å) (a, b, c)	73.7, 73.7, 332.2	50.9, 50.9, 322.8
Cell angles (º) (α, γ, β)	90, 90, 90	90, 90, 120
Completeness (%)	99.7 (98.2)	98.7 (99.9)
Total no. of reflections	322 715 (51 351)	740 569 (123 886)
Unique reflections	40 863 (6458)	48 590 (7787)
Redundancy	7.9 (8.0)	15.2 (15.9)
R_meas_ (%)	8.1 (111.7)	6.8 (115.4)
CC_1/2_ (%)	99.9 (84.9)	100.0 (93.5)
I/σ	15.6 (1.54)	22.05 (1.84)
Wilson B (Å^2^)	91.4	39.84
**Refinement statistics**		
R_work_/R_free_ (%)	22.99/28.51	22.06/25.52
No. of atoms		
Protein	4212	3263
Water	-	212
Cd^2+^	7	-
SCN	-	6
Na^+^		1
B factors (Å^2^)		
Chain A	126.0	41.8
Chain B	106.2	43.2
Water	-	44.9
Cd^2+^	167.1	-
SCN	-	59.0
Na^+^	-	33.8
R.m.s. deviations		
Bond lengths (Å)	0.005	0.012
Bond angles (º)	0.807	1.214
Validation		
MolProbity score	1.92	1.40
Clashscore	11.13	4.33
Poor rotamers	0	0.3
Ramachandran plot		
Favoured (%)	94.8	96.86
Outliers (%)	0	0.26

The crystal structure of Pf12-Pf41 shows that both proteins interact through two distinct binding sites (Site 1 and Site 2) with a total surface area of 1051 Å^2^ (Fig. 1C), with Site 1 and Site 2 contributing to the interaction surface with 578 Å^2^ and 473 Å^2^ respectively (Fig. 1C). Site 1 shows that the interdomain region of Pf12 forms contacts with the Pf41 D2 domain, with residues on both Pf12 domains interacting with extended loops on one side of the Pf41 D2 domain (Fig. 1B, C and D*top*). The Site 1 interface involves three potential salt bridges, several hydrogen bonds, π-π, as well as Met-π interactions (Fig. 1D*top*). Site 2 comprises of the Pf12 D2 domain in which its bottom β-sheet and surrounding loops form a concave protein surface that engages with the long α-helix of the Pf41 ID (Fig. 1B, C, and D *bottom*). Here, residues located along the Pf12 β-sheet form a hydrophobic network with residues located at the long α-helix of Pf41 ID (Fig. 1D*bottom*).

The Pf12 molecule in complex with Pf41 overlays with the existing unbound Pf12 structure (PDB ID 2YMO) with a root mean square deviation (r.m.s.d.) of 2.5 Å (over 1568 atoms), while the individual 6-cys domains align with r.m.s.d. values of 0.99 Å for D1 (over 570 atoms) and 0.58 Å for D2 (over 728 atoms). This finding indicates that the two 6-cys domains adopt different orientations towards each other in the bound and unbound state and provides evidence for potential mobility between the two domains in solution (Fig. 1E). Residues 84 to 116, which comprise β-strands β5 to β7 in the Pf12 D1 domain are not resolved in the unbound Pf12 structure but are well resolved in our crystal structure of the complex and shown to be involved in binding the Pf41 D2 domain (Fig. 1E). Bound and unbound Pf41 align with a r.m.s.d. of 0.73 Å over the range of 1342 atoms (Fig. 1D*right*), with the main difference being that large regions of the Pf41 ID are resolved in the presence of Pf12.

### Characterization of residues critical for Pf12-Pf41 complex formation

**Figure 2.**
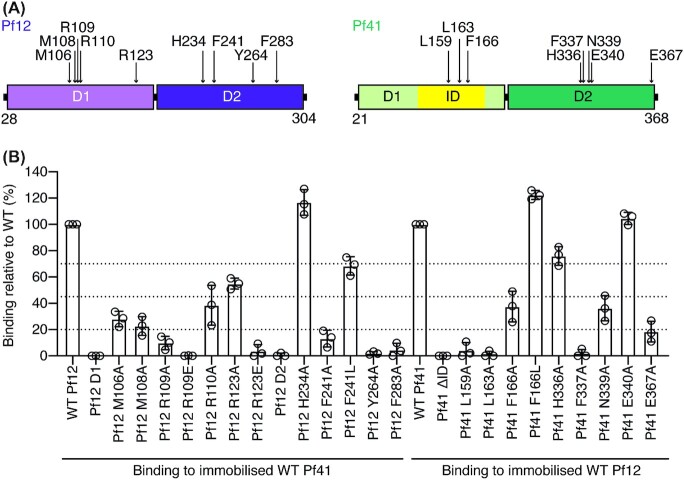
Structure-function analysis of Pf12 and Pf41 residues involved in complex formation. **(A)** Schematic diagram of recombinant proteins Pf12 D1D2 and Pf41 D1D2 with the mutated interfacing residues indicated with arrows. **(B)** Effect of mutations on Pf12–Pf41 complex formation. BLI-measurements between Pf12 and Pf41 wildtype and mutant proteins. Relative maximum response of each mutant at 1000 nM, 500 nM, and 250 nM concentration (represented by the dots) compared to wildtype protein maximum response is shown. Wildtype–wildtype binding was assigned to 100%. Dotted lines indicate loss of binding (<20% binding), strong loss of binding (21%–45%), intermediate loss of binding (46%–69%) and similar or no loss of binding (>70%) compared to wildtype. Bars represent the mean ± standard deviation (SD) for the three concentrations.

We used bio-layer interferometry (BLI) to further determine the role of the individual 6-cysteine domains in Pf12 or Pf41 in complex formation (Fig. 2). The single domain proteins Pf12 D1 and Pf12 D2 contain only the N-terminal D1 or the C-terminal D2 domain of Pf12, respectively (Fig. 2A). Pf41ΔID lacks the ID region and is replaced with a short glycine-serine linker (Parker et al. 2015), which has been shown previously to result in an abolishment of complex formation and will serve as a negative control. Pf12 D1 and Pf12 D2, as well as Pf41ΔID showed no binding to Pf41 D1D2 or Pf12 D1D2 respectively, suggesting that both binding sites are required for Pf12-Pf41 complex formation (Fig. 2B and S3 Fig.).

We performed site-directed mutagenesis to identify critical residues at the Pf12–Pf41 interface required for complex formation (Fig. 2A). The mutant proteins were expressed and purified and showed similar properties to wildtype in terms of expression level, elution in SEC and migration on SDS-PAGE (S2 and S3 Figs).

We examined the mutant Pf12 D1D2 and Pf41 D1D2 proteins for their ability to bind their respective wildtype interaction partner using BLI (Fig. 2B and S4 Fig.). To discuss the impact of each mutation, we present the relative maximum signal of each mutant compared to wildtype-wildtype binding (Fig. 2B). Calculated binding kinetic and affinity values are included in S4 Fig. At Site 1, we mutated Pf12 residues M106, M108, R109, R110, R123, H234, and Y264, and the Pf41 residues H336, F337, N339, E340, and E367 to alanine. We selected these residues as they are either involved in salt bridges, Arg-Met-Phe motifs or π-π interactions. H234 and H336 were mutated as they coordinate a cadmium ion at the Pf12-Pf41 interface, which we propose may feature a crystallization artefact rather than required for Pf12-Pf41 binding (Fig. 1D*top*). To further confirm if the Pf12 residues R109 and R123 were involved in salt bridge formation, we mutated both residues to glutamate to abolish the respective salt bridge and introduce an electrostatic repulsion at the Pf12-Pf41 interface.

For Site 1, Pf12 Y264A and Pf41 F337A did not form a complex with wildtype Pf41 and Pf12 respectively indicating the importance of the π-π interaction as critical for complex formation (Figs 1D*top*, 2B and Fig. S4). F337 is also involved in two Arg-Met-Phe motifs, with R123-M106 and R110-M108 respectively. The mutations R123A, M106A, R110A, and M108A of Pf12 reduced binding to Pf41 suggesting their contribution to the Pf12-Pf41 interaction (Figs 1D*top*, 2B and Fig. S4). The Pf41 mutant N339A had a decreased binding signal to Pf12 compared to wildtype protein which indicates that the hydrogen bond formed between N339 and the crucial Pf12 residue Y264 contributes to the complex formation of the two proteins. As expected, the mutation of H234 and H336 of Pf12 and Pf41 respectively, showed no significant decrease in protein-protein binding and therefore the ion-coordination at the contact site is not important for the Pf12-Pf41 interaction.

We examined the importance of the salt bridge formation at Site 1. While Pf12 mutant R123A reduced binding to wildtype protein, R123E was defective in binding to Pf41 highlighting the additional effect of the electrostatic repulsion (Figs 1D*top-left* and 2B). Pf41 residue E367 has the potential to form a salt bridge with Pf12 R123 and K62 (Fig. 1D*top-left*) and mutant E367A showed a diminished binding signal to Pf12 D1D2 compared to the Pf41 wildtype protein (Fig. 2B). Another potential salt bridge is formed between Pf41 residue E340 and Pf12 residue R109 (Fig. 1D*top-right*). The distance between them is relatively large, ∼ 5.5 Å. E340A showed a strong binding signal to Pf12 D1D2 suggesting that the potential interaction is not of importance. The guanidino group of Pf12 residue R109 is in closer proximity to other Pf12 residues, such as the side chain of E283 (∼ 4.6 Å) and carbonyl groups of P79 and T80 (∼ 3.2 Å and 3.3 Å, respectively). The reduced binding signal of R109A and defective binding of R109E to Pf41 might suggest a more important role of R109 in stabilizing the Pf12 region around β-strands β5-β7.

At Site 2, we mutated Pf12 residues F241 and F283, and Pf41 residues L159, L163, and F166 to alanine to examine the hydrophobic network between the Pf12 D2 domain and the long α-helix of Pf41. In addition, the phenylalanine residues were mutated to leucine to investigate the contribution of their aromatic character. Pf12 mutant F283A and Pf41 mutants L159A and L163A were defective in complex formation (Fig. 2B). F241 L of Pf12 and F166 L of Pf41 had no or only minor effects to the binding signal compared to wildtype protein (Fig. 2B), while the mutation to alanine showed a strong reduction of binding and indicates that the aromatic-aromatic interaction between Pf41 F166 and Pf12 F241/F283 is of less importance.

Our structure-function analysis identified critical residues of Pf12 and Pf41 for complex formation and highlights key interactions on both binding sites. Important for the assembly of Pf12 and Pf41 is a π-π interaction involving Pf12 residue Y264 and Pf41 residue F337, and hydrophobic contacts between Pf12 F283 and Pf41 residues L159 and L163. We find that both binding sites harbor crucial residues for the interaction between Pf12 and Pf41.

### Nanobodies against Pf12 and Pf41

**Figure 3.**
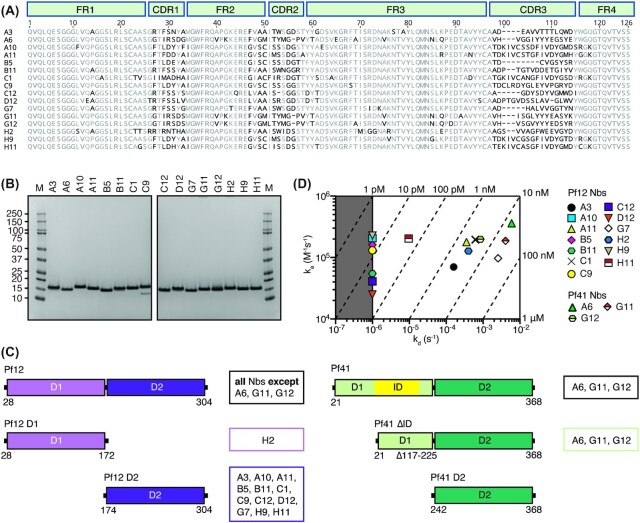
Pf12 and Pf41 specific nanobodies. **(A)** Sequence alignment of 16 nanobodies with framework regions (FR) and complementary determining regions (CDR) indicated according to the international ImMunoGeneTics information system (IMGT). Residues that represent less than 60% similarity to the consensus sequence are shown in black. **(B)** Coomassie-stained SDS-PAGE gels of purified nanobodies under reducing conditions. Molecular weight marker (M) in kDa is shown on the left. **(C)** Domain mapping of Pf12- and Pf41-specific nanobodies using BLI. Pf12 D1 and Pf12 D2 were examined for binding to anti-Pf12 nanobodies (*left*). Pf41 D2 and Pf41 ΔID, were tested for binding to anti-Pf41 nanobodies (*right*). Summary of nanobodies that recognise the respective protein domains are shown on the right of each schematic. **(D)** Iso-affinity plot showing the association rate constants (ka) vs dissociation rate constants (kd) of Pf12 or Pf41 binding to their respective nanobodies. Diagonal lines indicate equilibrium dissociation rate constants (*K*_D_). The area of the plot highlighted in grey indicates the detection limit of the instrument.

A nanobody phage display library of 10^8^ nanobodies was constructed from an alpaca immunised with Pf12-Pf41 complex. After two rounds of phage display, we identified 16 distinct nanobody clonal groups based on amino acid sequence homology of their complementary determining region 3 (CDR3) (Fig. 3A). We grouped nanobodies with identical CDR3 length and up to three amino acid differences into one clonal family. The CDR3 regions of the nanobodies vary in length between 9–17 residues (Fig. 3A). One member of each clonal group was expressed and purified, and referred to as A3, A6, A10, A11, B5, B11, C1, C9, C12, D12, G7, G11, G12, H2, H9, and H11. The 16 different nanobodies migrated between 15 and 17 kDa under reducing conditions (Fig. 3B).

We determined the binding selectivity of the nanobodies for Pf12 and Pf41 using BLI (Fig. 3C and Fig. S5). Thirteen nanobodies showed binding only to Pf12 D1D2, namely, A3, A10, A11, B5, B11, C1, C9, C12, D12, G7, H2, H9, and H11. All Pf12-specific nanobodies, except for H2, recognised Pf12 D2 whereas H2 bound only the Pf12 D1 domain (Fig. 3C*left* and S6 Fig). The three remaining nanobodies; A6, G11 and G12 bound Pf41 D1D2 (Fig. 3C*right* and Fig. S5). Furthermore, all three Pf41-specific nanobodies bound to Pf41ΔID but not Pf41 D2, indicating that these nanobodies recognise an epitope within the D1 domain of Pf41 but not within the ID (Fig. 3C*right* and Fig. S6).

We determined binding kinetics and affinities of the interaction between the nanobodies and Pf12 D1D2 or Pf41 D1D2, respectively using BLI (Fig. 3D and Fig. S5 and Table S2). The three anti-Pf41 nanobodies have binding affinities in the mid-to-low nanomolar range with equilibrium dissociation constants (*K*_D_) between 4 and 22 nM. Pf12-specific nanobodies bind their antigen with *K*_D_ values ranging from pico-to-mid nanomolar (<10 pM and 25 nM). Nanobody G7 is the Pf12-specific nanobody with the weakest affinity with a *K*_D_ of ∼25 nM. Four anti-Pf12 nanobodies (A10, B5, C9, and H9) have *K*_D_ values < 10 pM, which is below the detection limit.

### Pf12-specific nanobodies inhibit Pf12-Pf41 complex formation

**Figure 4.**
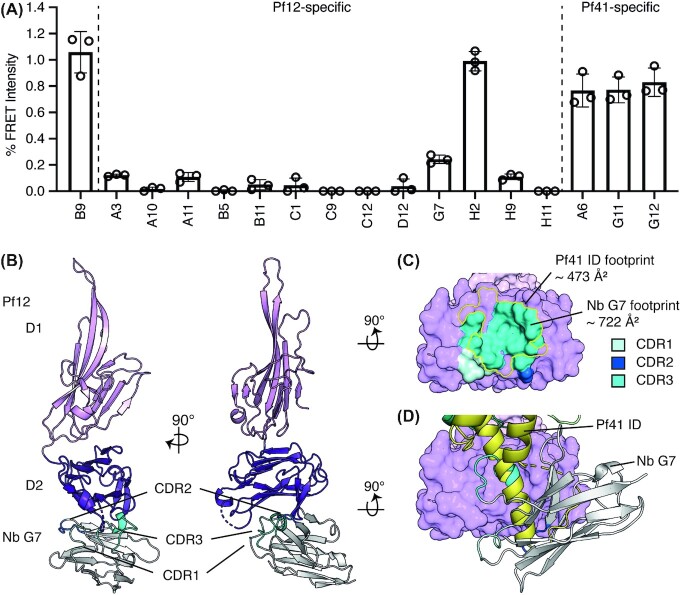
Pf12 specific nanobodies can inhibit Pf12-Pf41 complex formation. **(A)** Anti-Pf12 nanobodies inhibit Pf12-Pf41 complex formation in a FRET-based assay. The FRET signal is relative ‘no nanobody’ control. B9 is a nanobody specific for 6-cys protein Pf12p and does not recognise Pf12 or Pf41 (Dietrich et al. 2021). Nanobodies specific for Pf12 and Pf41 are indicated. Error bars represent standard error of the mean calculated from three independent measurements. Open circles represent the individual replicates. **(B)** Crystal structure of inhibitory Nb G7 in complex with Pf12 shown in two orthogonal views. All three CDR loops of Nb G7 (backbone, grey; CDR loops, blue) binds to the D2 domain of Pf12 (dark purple). **(C)** The Nb G7 epitope overlaps with the **Pf41 ID binding site on Pf12**. Pf12 is shown in surface representation (purple). The footprint of the three CDR loops of G7 are coloured in three different shades of blue and the footprint of Pf41 is indicated with a yellow outline. **(D)** Steric hindrance of the Pf12 D2 binding site. Secondary-structure matching (SSM) of Pf12 bound to Pf41 and Pf12 bound to Nb G7 based on the Pf12 D2 domains. Pf12 of Pf12-Nb G7 is shown in surface representation. Nb G7 (grey) and the insertion domain ID of Pf41 (yellow, shown in ribbon representation) clash as both proteins occupy a similar space around the Pf12 D2 domain.

Using a fluorescence resonance energy transfer (FRET)-based assay, we wanted to investigate if the nanobodies were able to disrupt Pf12-Pf41 complex formation (Fig. 4A). The resulting FRET signal in the absence of nanobody was arbitrarily designated as 100% FRET signal. The addition of an unrelated nanobody control, B9 did not affect Pf12-Pf41 complex formation whereas Pf12 specific nanobodies A10, B5, B11, C1, C9, C12, D12, and H11 completely abolished Pf12-Pf41 complex formation (Fig. 4A). Furthermore, G7 reduced the signal by ∼ 80%, and A3, A11 and H9 reduced it by ∼ 90%. Pf12 D1-specific nanobody H2 had no effect on the Pf12-Pf41 interaction and the three Pf41-specific nanobodies A6, G11, and G12 decreased the signal by only ∼20% (Fig. 4A).

### Crystal structure of Pf12 in complex with nanobody G7

Using X-ray crystallography, we determined the crystal structure of Pf12 D1D2 bound to nanobody G7 to a resolution of 1.77 Å (Fig. 4B and Table 1). Nanobody G7 binds to the bottom of the Pf12 D2 domain and not to the D1 domain, which is consistent with our mapping results (Fig. 3C). All three CDR loops of nanobody G7 form contacts with Pf12 residues and cover an interface area of ∼ 722 Å^2^, with CDR3 providing the major contribution to the paratope (Fig. 4B and C, and Table S1). The CDR3 loop forms a short α-helix, which bound into a hydrophobic cavity formed by the bottom β-sheet of the Pf12 D2 domain (Fig. 4B). This nanobody G7 epitope overlaps with the binding site of the Pf41 ID and therefore clash for simultaneous binding to Pf12 (Fig. 4C and D). Interestingly, the Pf12 residue F283, which is crucial for Pf41 binding is also engaged in binding to G7. Here, F283 is involved in hydrophobic contacts with residues H100 and V103 of the short CDR3 α-helix of G7 (S1 Table).

### Pf12- and Pf41-specific nanobodies show no effect on parasite egress and invasion in the blood stage of infection

Pf12 and Pf41 are readily detectable on the surface of merozoites suggesting that they could play an important role in either egress or invasion (Sanders et al. 2005, Gilson et al. 2006, Taechalertpaisarn et al. 2012). To test whether Pf12- and Pf41-specific nanobodies disrupted egress and/or invasion, schizont stage 3D7 Hyp1-Nluc parasites were exposed to increasing concentrations of the 16 nanobodies (up to 1 mg/ml), for four hours. As the schizonts rupture during this period, the bioluminescent Hyp1-Nluc protein which is exported into the erythrocyte compartment, is released into the growth media and its bioluminescence measured as a marker of merozoite egress (Dans et al. 2020). None of the nanobodies significantly reduced egress in comparison with the protein kinase G inhibitory compound ML10, which strongly impairs egress (Fig. S7). We also observed no reduction in invasion in the presence of nanobodies as compared to the complete inhibition of invasion in the presence of heparin (Fig. S7).

## Materials and methods

### Cloning, protein production and purification

Recombinant fragments of Pf12 D1D2 (residues N28-S304) and Pf41 D1D2 (residues K21-S368) were produced and purified as described previously with the following modifications (Dietrich et al. 2021). Briefly, the genes were codon optimised for insect cells (Genscript) and the corresponding fragments amplified by PCR and cloned into a modified pAcGP67-A vector via restriction-ligation cloning using NheI-HF and NotI-HF restriction enzymes (NEB). The Pf12 construct lacks the signal peptide and the GPI-anchor site and the Pf41 construct lacks the signal peptide and ten C-terminal residues. Both constructs are expressed with the secretion signal sequence gp67, and an N-terminal TEV-protease cleavable octa-histidine tag leaving three additional residues, GAS, as cloning artefacts. All *N*-glycosylation sites were kept intact.

Mutant gene sequences were either synthesised and subcloned into the expression vector (modified pAcGP67-A) by IDT or synthesised by Genscript with NheI-HF and NotI-HF restriction enzyme sites, subcloned into the expression vector in-house and sequenced for confirmation. Wildtype and mutant proteins, as well as single domain Pf12 (D1 residues N28-L172 and D2 residues E174-S304) and Pf41 constructs (D2 residues I242-S368) were purified via the same purification protocol. A summary of the different mutants and their sequences can be found in the supplementary.

*Spodoptera frugiperda* 21 cells were used for the generation and amplification of expression viruses as well as for protein production. After three days of infection, growth media was harvested, supplemented with PMSF and a cOmplete protease inhibitor tablet (Roche) and dialysed against 30 mM Tris pH 7.5, 300 mM NaCl. The proteins were purified via Ni-IMAC. The octa-histidine tag was removed by TEV protease cleavage while dialysing into 30 mM Tris pH 7.5, 300 mM NaCl and a following Ni-IMAC. Size exclusion chromatography (SEC) in 20 mM HEPES pH 7.5, 150 mM NaCl was used as a last purification step. In addition, wildtype proteins were also purified as tagged proteins via Ni-IMAC following SEC (SD200 increase 10/300, Cytiva).

### Pf12-Pf41 and Pf12-Nb complex formation and de-glycosylation using PNGase F

Purified, untagged Pf12 D1D2 and Pf41 D1D2 proteins were mixed in a molar ratio of 1.2:1 and incubated for 1 h on ice. SEC in 20 mM HEPES pH 7.5, 150 mM NaCl was performed to separate the formed Pf12-Pf41 complex from excess Pf12. For the purpose of de-glycosylation Glyco buffer 2 and PNGase F (NEB) was added to the mix of Pf12 and Pf41, incubated at RT for 1h prior SEC in 20 mM HEPES pH 7.5, 150 mM NaCl.

Purified, untagged Pf12 D1D2 was mixed with Glyco buffer 2 and PNGase F (NEB) and incubated at RT for 1h prior the addition of Nb in a molar ratio of 1:1.5. The proteins were incubated for 1h on ice followed by SEC in 20 mM HEPES pH 7.5, 150 mM NaCl to separate the formed Pf12-Nb complex from excess Nb.

### Crystallization and structure determination

Initial crystallization trials were performed at the Collaborative Crystallization Centre (CSIRO, C3, Parkville) at 8ºC. Purified Pf12-Pf41 complex with intact glycosylation crystallised as fuzzy needle clusters in 5 mM cadmium chloride, 20% (w/v) PEG 4000, 50 mM HEPES pH 7.5. Complex treated with PNGase F crystallised in the same condition as thin plates. Crystals appeared overnight. Hanging-drop vapour diffusion experiments were set up in-house. Three-dimensional crystals grew in 10 mM cadmium chloride, 14% (w/v) PEG 4000, 25% glycerol, 50 mM HEPES pH 7.5 and were further optimized by micro-seeding. Pf12-Pf41 crystals were harvested from mother liquor and flash frozen in liquid nitrogen. Pf12 in complex to Nb G7 crystallized in 15% (w/v) PEG 4000, 200 mM potassium thiocyanate, 0.05 M sodium cacodylate pH 6, were flash frozen with 30% glycerol in mother liquor. Diffraction data was collected at the MX2 beamline at the Australian synchrotron. The XDS package was used for data processing. The phase problem was solved by molecular replacement using the program Phaser. The atomic coordinates of Pf12 D1D2 (PDB ID 2YMO) and Pf41 D1D2 (PDB ID 4YS4) were used as search models for the Pf12-Pf41 complex structure. PDB ID 2YMO and the coordinates of Nb B5 of PDB ID 7KJH were used to solve the crystal structure of Pf12 bound to Nb G7. Anomalous data collected on Pf12-Pf41 crystal was used to confirm the presence of bound Cd-ions. Alternating rounds of structure building and refinement were carried out using *Coot* and phenix (Adams et al. 2002, Emsley et al. 2010, Afonine et al. 2012). About 2000 reflections were set aside for the calculation of R_free_. As parts of the of Pf41 ID were not directly connected to the remaining Pf41 protein, the map sharpening tool of *Coot* was used to assist with building this region. All figures of the structures were prepared with PyMOL (www.pymol.com) and interactions, interfaces and buried areas from solvent were analysed using PISA (Krissinel 2015). The atomic coordinates and structure factor files have been deposited in the Protein Data Bank under the accession codes 7S7Q for Pf12-Pf41 and 7S7R for Pf12-G7.

### Mutational analyses using BLI

Protein interactions of Pf12 and Pf41 mutants were performed on the Octet RED96e (FortéBio). All assays were performed using NiNTA capture sensor tips (NTA) sensors (FortéBio) with kinetics buffer (PBS pH 7.4 supplemented with 0.1% (w/v) BSA and 0.05% (v/v) TWEEN-20) at 25°C. After a 60 s biosensor baseline step, octa-histidine tagged Pf12 or Pf41 (20 μg/mL) were loaded to a signal of 1.8 nm onto NTA sensors by submerging sensor tips for 200 s and then washed in kinetics buffer for 60 s. Association of Pf12 mutants to Pf41 was observed using a two-fold dilution series of untagged Pf12 mutant from 31–1000 nM for 180 s and dissociation was observed in kinetics buffer for 180 s. Association of Pf41 mutants to Pf12 was observed using the same conditions. Sensor tips were regenerated using a cycle of 5 s in 300 mM imidazole pH 7.5 and 5 s in kinetics buffer repeated five times. Baseline drift was corrected by subtracting the response of a protein loaded sensor not incubated with untagged mutant protein. We observed a fast and slow association rate of the Pf12-Pf41 interaction; therefore, we used a global fit 2:1 heterogeneous model for the curve fitting analysis to determine K_D_ values and kinetic parameters. This analysis was performed using Octet Data Analysis 10.0 software. We hypothesise that the two separate interaction sites and flexibility of the regions involved in Pf12-Pf41 binding result in a complex association kinetic rate that is better modelled with a heterogeneous 2:1 binding model rather than a 1:1 binding model. Curves that could not be fitted were excluded from the analyses. The maximum response for each mutant relative to wildtype at concentrations of 1000 nM, 500 nM, and 250 nM were calculated by dividing the maximum response of a mutant protein by the maximum response of the wildtype protein at each concentration. The relative maximum response at each concentration was plotted with the mean ± standard deviation (SD).

### Nanobodies affinities using BLI

Affinity measurements were performed on the Octet RED96e using the above method with the following modifications. After a 60 s biosensor baseline step, nanobodies (5 μg/mL) were loaded onto NTA sensors by submerging sensor tips until a response of 0.5 nm and then washed in kinetics buffer for 60 s. Pf12 and Pf41 association measurements were performed using a two-fold dilution series of untagged Pf12 or Pf41 from 6–200 nM for 180 s and dissociation was measured in kinetics buffer for 180 s. Sensor tips were regenerated using a cycle of 5 s in 300 mM imidazole pH 7.5 and 5 s in kinetics buffer repeated five times. Baseline drift was corrected by subtracting the response of a nanobody loaded sensor not incubated with untagged protein. Curve fitting analysis was performed with Octet Data Analysis 10.0 software using a global fit 1:1 model to determine K_D_ values and kinetic parameters. Curves that could not be fitted were excluded from the analyses. Mean kinetic constants reported are the result of two independent experiments.

### Alpaca immunisation and nanobody phage library

One alpaca was subcutaneously immunised six times with approximately 200 μg of recombinant glycosylated Pf12-Pf41 complex. The adjuvant used was GERBU FAMA. Immunization and handling of the alpaca for scientific purposes was approved by Agriculture Victoria, Wildlife & Small Institutions Animal Ethics Committee, project approval No. 26–17. Blood was collected three days after the last immunization for the preparation of lymphocytes. Nanobody library construction was carried out according to established methods (Pardon et al. 2014). Briefly, alpaca lymphocyte mRNA was extracted and amplified by RT-PCR with specific primers to generate a cDNA library size of 10^8^ nanobodies with 80% correct sized nanobody insert. The library was cloned into a pMES4 phagemid vector amplified in *Escherichia coli* TG1 strain and subsequently infected with M13K07 helper phage for recombinant phage expression.

### Isolation of nanobodies against Pf12-Pf41

Biopanning for anti-Pf12-Pf41 nanobodies using phage display was performed as previously described with the following modifications (Pardon et al. 2014). Phages displaying Pf12-Pf41-specific nanobodies were enriched after two rounds of biopanning on 1 μg of immobilised Pf12-Pf41 complex. After the second round of panning, 95 individual clones were selected for further analyses by ELISA for the presence of Pf12-Pf41 nanobodies. Positive clones were sequenced and annotated using the International ImMunoGeneTics database (IMGT) and aligned in Geneious Prime (Brochet et al. 2008).

### Expression and purification of nanobodies

Nanobodies were expressed in *E. coli* WK6 cells. Bacteria were grown in Terrific Broth at 37°C to an OD_600_ of 0.7, induced with 1 mM IPTG and grown overnight at 28°C for 16 h. Cell pellets were harvested and resuspended in 20% sucrose, 20 mM imidazole, 150 mM NaCl DPBS, and incubated for 15 min on ice. 5 mM EDTA pH 8.0 was added and incubated on ice for 20 minutes. After this incubation, 10 mM MgCl_2_ was added, and periplasmic extracts were harvested by centrifugation and the supernatant was loaded onto a 1 ml HisTrap FF column (GE Healthcare). The nanobody was eluted via a linear gradient into 400 mM imidazole, 100 mM NaCl, PBS. The appropriate fractions were concentrated and subjected to SEC (SD200 increase 10/300) pre-equilibrated in 20 mM HEPES pH 7.5, 150 mM NaCl.

### Analytical ultracentrifugal analysis

Samples were analysed using an XL-I analytical ultracentrifuge (Beckman Coulter, Indianapolis, USA) equipped with an AnTi-60 rotor. Protein samples were loaded in the sample compartment of double-sector epon centrepieces, with buffer in the reference compartment. Radial absorbance data was acquired at 20°C using a rotor speed of 50 000 rpm and a wavelength of 280 nm, with radial increments of 0.003 cm in continuous scanning mode. The sedimenting boundaries were fitted to a model that describes the sedimentation of a distribution of sedimentation coefficients with no assumption of heterogeneity (c(s)) using the program SEDFIT (Schuck 2000). Data were fitted using a regularization parameter of p  = 0.95, floating frictional ratios, and 100 sedimentation coefficient increments in the range of 0–15 S.

### FRET assay

Pf12 was labelled with N-hydroxysuccinimide ester-activated DyLight 488 (DL488) and Pf41 labelled with DyLight 594 (DL594) (Life Technologies). The dyes were dissolved in dimethyl sulfoxide (DMSO) (Sigma) before incubation with the proteins. Pf12 was incubated with DyLight 488 at a 4:1 molar ratio of dye to protein for 30 minutes at room temperature. Pf41 was incubated with DyLight 594 at a 1:1 molar ratio of dye to protein for one hour on ice. Un-conjugated dye was removed using a Zeba Spin Desalting column (7K MWCO) (ThermoFisher). Average dye per protein was ∼ 2.4 dye/protein for Pf12 and ∼ 1.0 dye/protein for Pf41. To test inhibitory nanobodies, Pf12-DL488 and Pf41-DL594 were mixed in a 1:1 molar ratio with five-fold excess of nanobody. The FRET assays were read in Corning 384-well plates and each sample was performed in triplicate. 5 μL of 0.2% SDS, 10% 2-mercaptoethanol in FRET buffer was added to one triplicate sample to denature the proteins and measure the background signal in the absence of complex formation. Fluorescence intensity was measured using EnVision plate reader (PerkinElmer Life Sciences). DL488 (donor) fluorescence was measured with a 485/14-nm excitation filter and 535/25-nm emission filter and DL594 (acceptor) fluorescence was measured with a 590/20-nm excitation filter and 615/9-nm emission filter. Sensitised emission was measured with a 485/14-nm excitation filter and 615/9-nm emission filter. Fluorescence measurements were analysed using Prism 6 software (GraphPad). To account for bleed-through of dyes into the sensitised emission spectra, standard curves for both dyes were measured and the raw data was transformed by multiplying with the slope of the standards. The y-intercept from the standard curves indicated the background fluorescence when no dye was present. The FRET signal was calculated with the following equation: FRET signal = Raw sensitised emission—transformed DL488 emission—transformed DL594 emission—(average of DL488 standard curve y-intercept and DL594 standard curve y-intercept). To compare between independent replicates, percentage FRET signal relative to the no inhibitor control was calculated by dividing the FRET signal with inhibitor by the FRET signal with no inhibitor, multiplied by 100.

### Parasite culturing

*Plasmodium falciparum* parasites were continuously cultured as previously described (Jensen and Trager 1977). Parasites were grown in human erythrocytes provided by the Australian Red Cross Blood Bank at 4% (v/v) haematocrit in complete RPMI medium (RPMI-1640 (Sigma), 25 mM HEPES (GIBCO), 0.37 mM hypoxanthine (Sigma), 31.25 µg/mL gentamicin (GIBCO), 0.2% (w/v) NaHCO_3_ (Thermo Scientific), 0.5% (w/v) AlbuMAX II (GIBCO)), at 37°C in low oxygen conditions (1% O_2_, 5% CO_2_, 94% N_2_). 3D7 Hyp1-Nluc parasites were used which episomally expresses the exported protein *Pf*Hyp1 (PF3D7_0 113 300) tagged with the bioluminescent reporter nanoluciferase (Nluc), under the control of *P. berghei ef1α* protomer (active from the trophozoite stage) (Azevedo et al. 2014). The Hyp1-Nluc plasmid was maintained by continuous exposure to 2.5 nM WR99210 (Jacobus).

### Egress and invasion assay

Parasites were synchronised by adding 25 nM egress inhibitor ML10 (a *Pf*PKG inhibitor; LifeArc) to trophozoites overnight. Next morning the ML10 was removed and the late stage schizonts were allowed to egress and invade for 4 h. The remaining schizonts were eliminated by sorbitol treatment and the new ring stage parasites were grown for nearly 2 days until late stage schizonts which were enriched from uninfected erythrocytes and younger parasites over a 67% Percoll density gradient. The egress/invasion assay was then performed as previously described (Dans et al. 2020). Briefly, the schizonts were mixed with uninfected erythrocytes to 1%–2% parasitemia in 1% haematocrit and were incubated in duplicate with a range of nanobody concentrations (from 0.004 to 1 mg/mL), with 40% (v/v) of the storing buffer alone (20 mM HEPES pH 7.5, 150 mM NaCl), with 25 nM ML10 (egress inhibitor control) or with 100 μg/mL heparin (invasion inhibitor control; Sigma). These cultures were incubated for 4 h at 37°C to permit egress and invasion. After this, 5 μL of the growth medium was retained to measure egress, the cultures were sorbitol treated to retain only newly formed rings, and 5 µL of whole culture was harvested 24 hours later to measure invasion. These samples were added to 45 μL of 1x Nano-Glo® Luciferase Assay Buffer containing 1:1 000 Nano-Glo® substrate (Promega) in a 96-well white opaque plate. Luminescence was measured on a CLARIOstar plate reader (BMG Labtech).

## Discussion

*Plasmodium* 6-cys proteins are expressed at different life cycle stages and are important for parasite growth, fertilization, transmission, and immune evasion. In this study, we present a 2.85 Å resolution crystal structure of the Pf12-Pf41 complex and extensive structure function analyses to identify critical residues required for complex formation. In addition, we identified Pf12 specific nanobodies that can inhibit Pf12-Pf41 complex formation. Using X-ray crystallography, we show that Pf12 specific Nb G7 blocks Pf12-Pf41 binding by steric hindrance.

Our crystal structure of the Pf12-Pf41 complex identified two critical interfaces, Site 1 and Site 2, which are required for complex formation. Previous studies which used cross-linking approaches to develop a model of this heterodimeric complex showed a few differences from our Pf12-Pf41 crystal structure (Parker et al. 2015). Like the cross-linking results, we verify an anti-parallel orientation of the two proteins towards each other. However, one of the main differences is that our crystal structure shows clearly that the Pf41 D2 domain engages Pf12 at a groove of the Pf12 D1-D2 domain junction, therefore forming contacts with both Pf12 domains. A second difference involves the Pf41 ID: the cross-linking model which proposes that the ID of Pf41 contacts both domains of Pf12, however our crystal structure of the Pf12-Pf41 complex reveals binding only between the Pf41 ID and the Pf12 D2 domain. Our crystal structure of the Pf12-Pf41 complex provides structural resolution of the Pf41 ID that was mostly absent from the crystal structure of Pf41 alone (Parker et al. 2015). The now resolved long α-helix of Pf41 ID from 152–178 forms a hydrophobic network with the Pf12 D2 domain. We show that the mutation of single residues at the Pf12 D2–Pf41 ID binding site (F283A for Pf12, L159A and L163A for Pf41) abolished complex formation.

Our crystal structure of the Pf12-Pf41 complex indicates that both Site 1 and Site 2 binding sites are important for complex formation. PISA analysis suggests that the heterodimeric complex is a stable biological assembly, and the two binding sites contribute to the interaction surface with 578 Å^2^ (Site 1, between the Pf12 interdomain region and Pf41 D2), and 473 Å^2^ (Site 2, between Pf12 D2 and Pf41 ID), and calculated solvation free energy gains of −10.3 and −11.0 kcal/mol, respectively. The calculated interaction areas and solvation free energy gains indicate that both binding sites contribute to a similar extent to the Pf12-Pf41 engagement. Furthermore, we show that neither Pf12 D2 nor Pf41ΔID were able to form a Pf12-Pf41 complex. These results indicate that the Pf41 ID interaction with Pf12 is necessary but not sufficient for complex formation and that both Site 1 and Site 2 are critical for the assembly of the two 6-cys proteins.

The crystal structure of the Pf12-Pf41 complex suggests that binding of Pf12 to Pf41 causes conformational changes within the proteins. We found that the D1 and D2 domains of bound and unbound Pf12 adopt different orientations towards each other. Furthermore, a region of 33 residues spanning 84–116, which includes the β-sheets β5 to β7 of the Pf12 D1 domain, which was unstructured in the unbound Pf12 molecule, was well resolved in the presence of Pf41, suggesting that binding of Pf41 possibly reduces the flexibility within Pf12 and stabilises the junction of the Pf12 D1-D2 domains. The Pf41 ID consists of only ∼ 40% secondary structure elements and its inherent flexibility is supported by higher B-factors of the non-coordinated regions and unresolved residues that connects it to the core structure of the 6-cys domain. The long α-helix which interacts with Pf12 has lower B-factors than the remaining parts of the Pf41 ID suggesting a stabilizing effect caused by Pf12-binding.

Using site directed mutagenesis, we observed that on both binding sites either aromatic or solely hydrophobic residues played crucial roles in the complex assembly. Alanine substitutions of Pf12 residues Y264 or F283 abolished binding to Pf41. On Pf41, alanine mutants of F337, L159, and L163 were defective for binding to Pf12. These residues, which appear critical for Pf12 and Pf41 binding, are also conserved in orthologous proteins and may also play critical roles in P12-P41 complex formation in other *Plasmodium spp* (Aurrecoechea et al. 2009).

All Pf12-specific nanobodies, except for H2, block Pf12-Pf41 complex formation. Furthermore, the inhibitory nanobodies bind to the Pf12 D2 domain, while the non-inhibitory H2 nanobody binds to the Pf12 D1 domain. The crystal structure of Pf12 in complex with inhibitory nanobody G7 showed that the CDR loops on G7 interact with the bottom β-sheet of the Pf12 D2 domain. The CDR3 loop folds into a small α-helix which binds to the Pf12 β-sheet that normally accommodates the long α-helix of the Pf41 ID. G7 binds to Pf12 with higher affinity compared to Pf12-Pf41 binding (25 nM vs 120 nM, (Dietrich et al. 2021)) and the nanobody epitope is larger at 722 Å^2^ compared to the ID binding site on Pf12, which comprises 473 Å^2^. We propose that G7 inhibits Pf12-Pf41 complex assembly by binding Pf12 with higher affinity and blocking the binding site for Pf41 ID.

The CDR3 loops of nanobodies are often longer compared to conventional antibodies, which allows them to bind to groove-like structures (Bannas et al. 2017). This is exemplified by G7 binding to the hydrophobic groove at the bottom of the Pf12 D2 domain. Additionally, we showed that nanobodies specific for Pf12p (which is the paralogue of Pf12), bound to this 6-cys protein at a groove-like region of the Pf12p-interdomain region (Dietrich et al. 2021). While we were unsuccessful in obtaining crystals with other Pf12-nanobody complexes, we propose that an epitope near the interdomain region of Pf12 might be another potential binding site of inhibitory nanobodies. All Pf41-specific nanobodies bound outside the ID and were non-inhibitory. In the future, direct selection of Pf41-specific nanobodies that bind to the ID could yield nanobodies that block complex formation between Pf12 and Pf41.

Our inhibitory nanobodies were able to block Pf12-Pf41 complex formation, but they did not inhibit egress or invasion of blood stage parasites *in vitro*. This is consistent with previous findings showing that Pf12 and Pf41 could be knocked-out without impacts in parasite growth (Taechalertpaisarn et al. 2012). One potential explanation is that Pf41 may recruit another parasite protein during invasion whose binding is not blocked by the nanobodies. Previous studies show that in the absence of Pf12, non-GPI anchored Pf41 remained on the parasite surface suggesting that Pf41 must interact with at least one other membrane-bound merozoite surface protein (Taechalertpaisarn et al. 2012). Furthermore, in *P. vivax*, another Pv12-interaction partner, PVX_110 945, was identified in an AVEXIS screen (Hostetler et al. 2015). Therefore, it is possible that Pf12 and Pf41 may bind to alternative parasite proteins for their roles in red blood cell invasion. Furthermore, Pf12 and Pf41 may play a different role in the parasite life cycle. Similar to merozoite protein Pf92 which is a highly abundant GPI-anchored 6-cys protein, the Pf12-Pf41 heterodimer might be involved in interactions with human complement regulators. Future experiments, such as complement destruction assays could be used to establish a potential function of these proteins in immune evasion. The nanobodies raised in this study will be useful to identify host interaction partners or alternative parasite binding proteins via co-immunoprecipitation experiments and might help to determine the function of Pf12 and Pf41 in the future.

## Supplementary Material

xtac005_Supplemental_Files
