# Supplementary material for: Structure of the Pf12 and Pf41 heterodimeric complex of Plasmodium falciparum 6-cysteine proteins
Source: FEMS Microbes. 2022 Feb 16;3:xtac005. doi: 10.1093/femsmc/xtac005 (PMC8930183; doi:10.1093/femsmc/xtac005)
Supplement: xtac005_Supplemental_Files [file xtac005_supplemental_files.zip › S1_and_S2_Tables.pdf]

S1 Table. Interactions between Pf12 and Pf41, and Pf12 and nanobody G7.

Pf12 and Pf41 (PDB ID 7S7Q)

Pf12	Group	Location	Pf41	Group	Location	Distance (Å)
			**Hydrogen bonds**			
Arg 109	N	D1	Asn 339	O	D2	2.9
Arg 110	NH1	D1	Pro 365	O	D2	3.8
Tyr 264	OH	D2	Leu 313	O	D2	2.6
Tyr 264	OH	D2	Asn 339	N	D2	3.2
Ser 287	OG	D2	Lys 170	NZ	ID	3.9
			**Salt bridges**			
Lys 62	NZ	D1	Glu 367	OE1	D2	3.8
Arg 123	NH1/NH2	D1	Glu 367	OE2	D2	2.9/3.7

**Other Pf12 interfacing residues**				**Other Pf41 interfacing residues**		
Glu 83	Met 106	Phe 107		Ala 155	Asp 156	Tyr 158
Met 108	Ser 111	Ile 117		Leu 159	Asn 160	Ala 162
Ser 121	Asp 208	Val 210		Leu 163	Arg 165	Phe 166
Thr 212	Glu 232	His 234		Lys 167	Met 169	Thr 312
Asp 235	Val 238	Phe 241		Ile 314	Pro 315	Gly 316
Leu 243	Ser 244	Gly 245		Tyr 317	Pro 335	His 336
Lys 246	Met 261	Asp 262		Phe 337	Val 338	Glu 340
His 263	Lys 281	Phe 283		Gln 341	Tyr 342	Gln 364
Val 285	Lys 293	Val 295		Gly 366		
Thr 297						

Pf12 and nanobody G7 (PDB ID 7S7R)

Pf12	Group	Location	Nb G7	Group	Location	Distance (Å)
			**Hydrogen bonds**			
Asp 208	O	D2	Tyr 32	OH	CDR1	2.8
Asn 279	ND2	D2	Ser 57	OG	CDR2	3.8
Lys 293	NZ	D2	Val 104	O	CDR3	2.8
			**Salt bridges**			
Lys 246	NZ	D2	Glu 44	OE1	FR2	3.1
Lys 281	NZ	D2	Asp 99	OD2	CDR3	2.7

			**Other Pf12 interfacing residues**			
Asn 207	Val 210	Thr 212		Thr 28	Ser 31	Gly 33I
Lys 214	Phe 241	Asn 242		Phe 37	Arg 45	Glu 46
Leu 243	Ser 244	Gly 245		Phe 47	Ala 50	Ser 52
Lys 246	Trp 280	Phe 283		Trp 53	Asp 56	Thr 58
Val 285	Val 295	Glu 299		Tyr 59	His 100	Leu 102
Ala 300	Ser 301			Val 103	Thr 107	Tyr 108
				Tyr 110		

Interactions and interfacing residues were determined using PISA (39).

S2 Table. Binding kinetics and affinity of Pf12 and Pf41 interactions with their specific nanobodies.

Pf12 Nb	K_D (nM)	k_a (x 10^5 $M^{-1}s^{-1}$)	k_d (x 10^{-5} s^{-1})
A3	2.29 (± 0.47)	0.70 (± 0.00)	15.95 (± 3.32)
A10	< 0.01	2.07 (± 0.05)	< 0.1
A11	2.00 (± 0.12)	1.79 (± 0.02)	35.60 (± 2.40)
B5	< 0.01	1.61 (± 0.03)	< 0.1
B11	< 0.01	0.55 (± 0.01)	< 0.1
C1	3.18 (± 0.08)	1.95 (± 0.06)	61.85 (± 3.32)
C9	< 0.01	1.31 (± 0.01)	< 0.1
C12	< 0.01	0.41 (± 0.01)	< 0.1
D12	< 0.01	0.25 (± 0.04)	< 0.1
G7	26.25 (± 1.63)	0.97 (± 0.01)	254.50 (± 17.68)
H2	3.17 (± 0.25)	1.28 (± 0.04)	40.20 (± 2.12)
H9	< 0.01	2.28 (± 0.08)	< 0.1
H11	0.05 (± 0.01)	2.04 (± 0.08)	0.96 (± 0.31)
**Pf41 Nb**	**K_D (nM)**	**k_a (x 10^5 $M^{-1}s^{-1}$)**	**k_d (x 10^{-5} s^{-1})**
A6	16.55 (± 0.92)	3.61 (± 0.26)	597.00 (± 9.90)
G11	21.70 (± 0.00)	1.88 (± 0.06)	406.00 (± 14.14)
G12	4.28 (± 0.40)	1.99 (± 0.06)	84.60 (± 5.37)
