# Supplementary figures and images for: Structure of the Pf12 and Pf41 heterodimeric complex of Plasmodium falciparum 6-cysteine proteins
Source: FEMS Microbes. 2022 Feb 16;3:xtac005. doi: 10.1093/femsmc/xtac005 (PMC8930183; doi:10.1093/femsmc/xtac005)

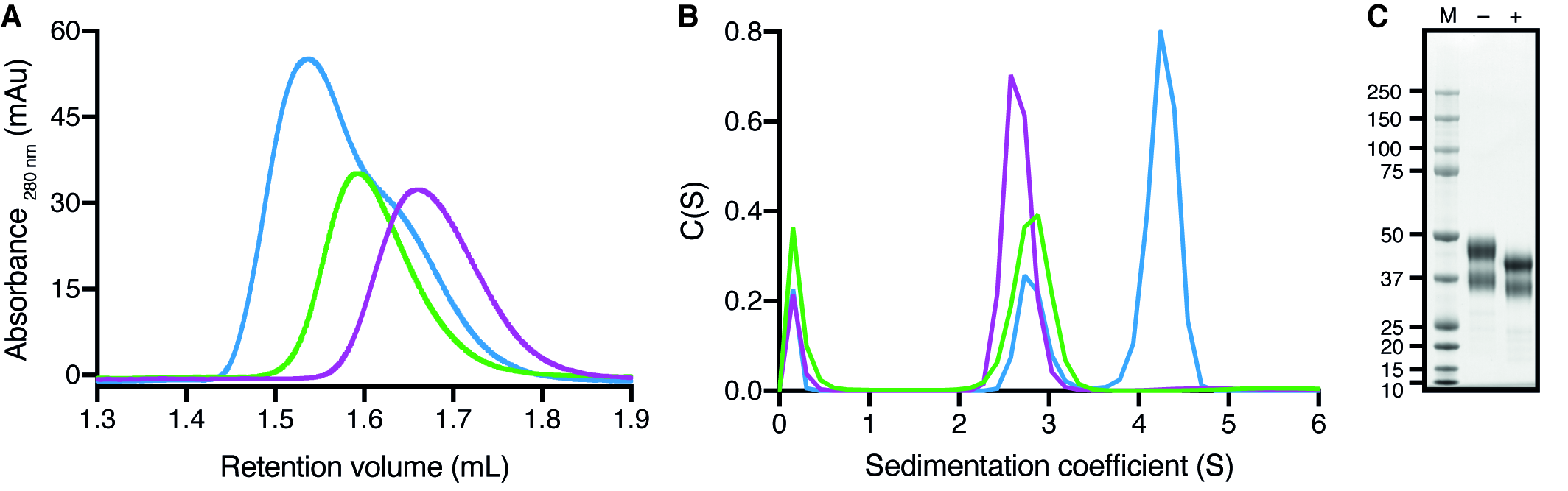

Supplement: xtac005_Supplemental_Files [file xtac005_supplemental_files.zip › S1Fig.tif]

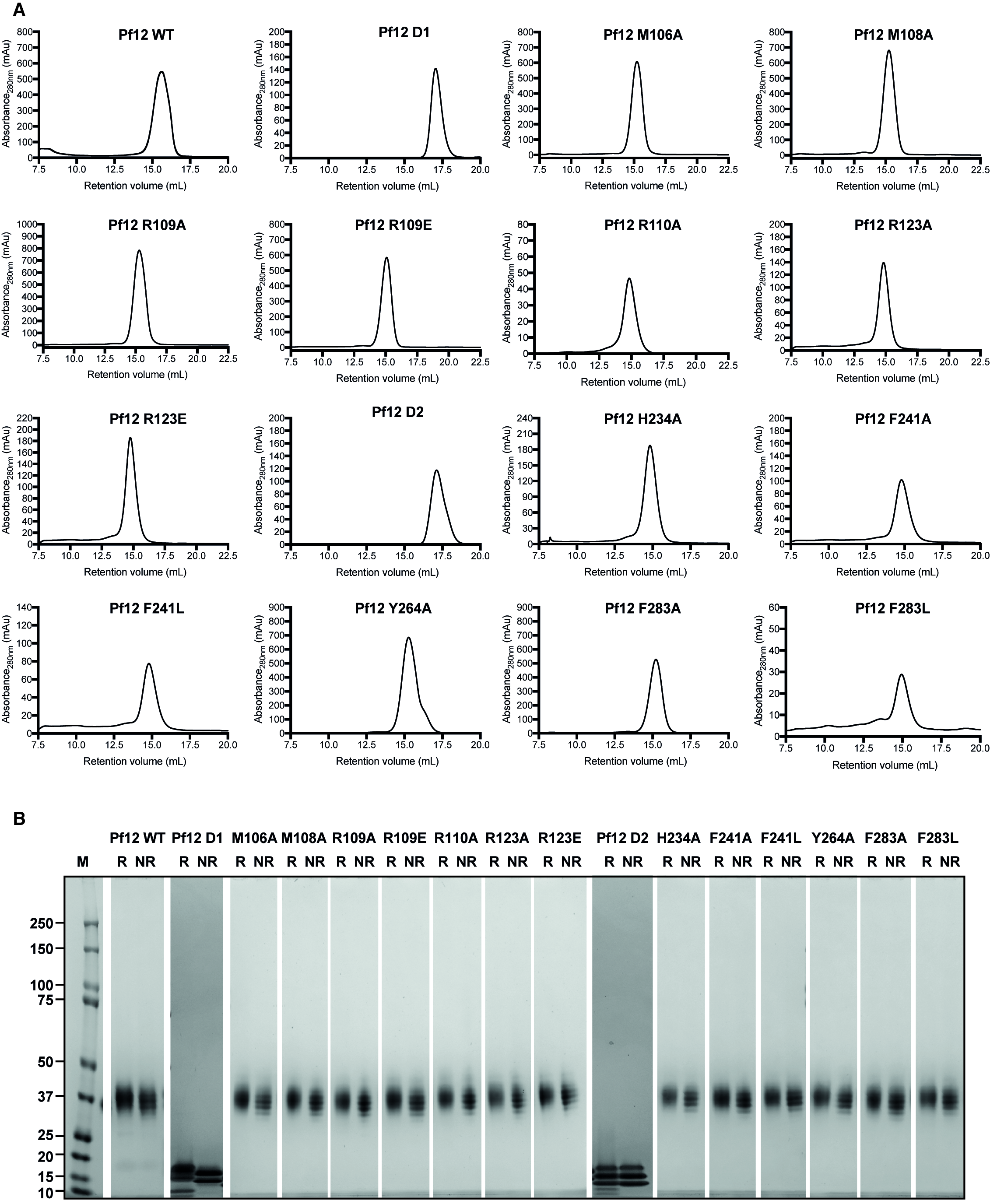

Supplement: xtac005_Supplemental_Files [file xtac005_supplemental_files.zip › S2Fig.tif]

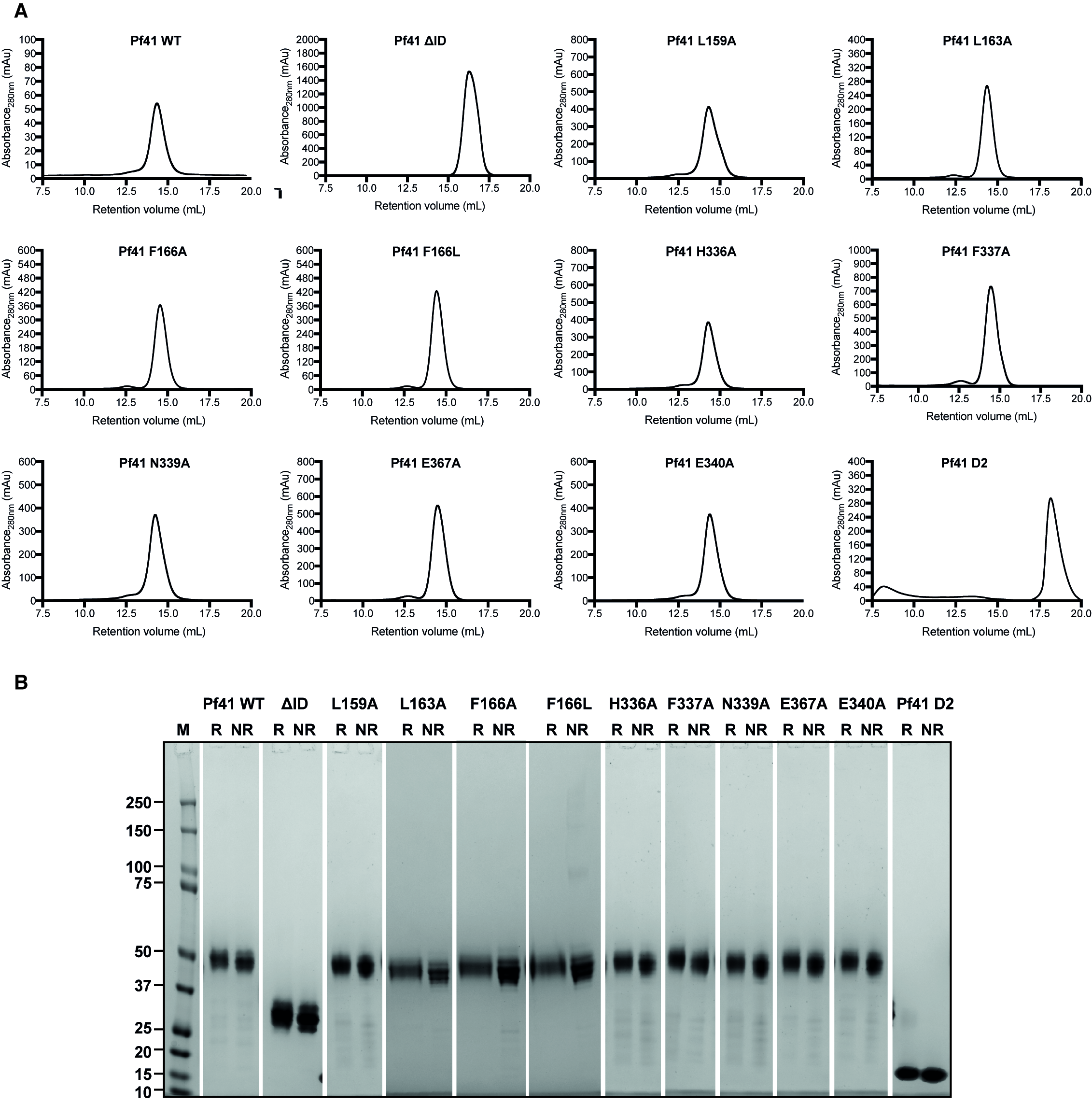

Supplement: xtac005_Supplemental_Files [file xtac005_supplemental_files.zip › S3Fig.tif]

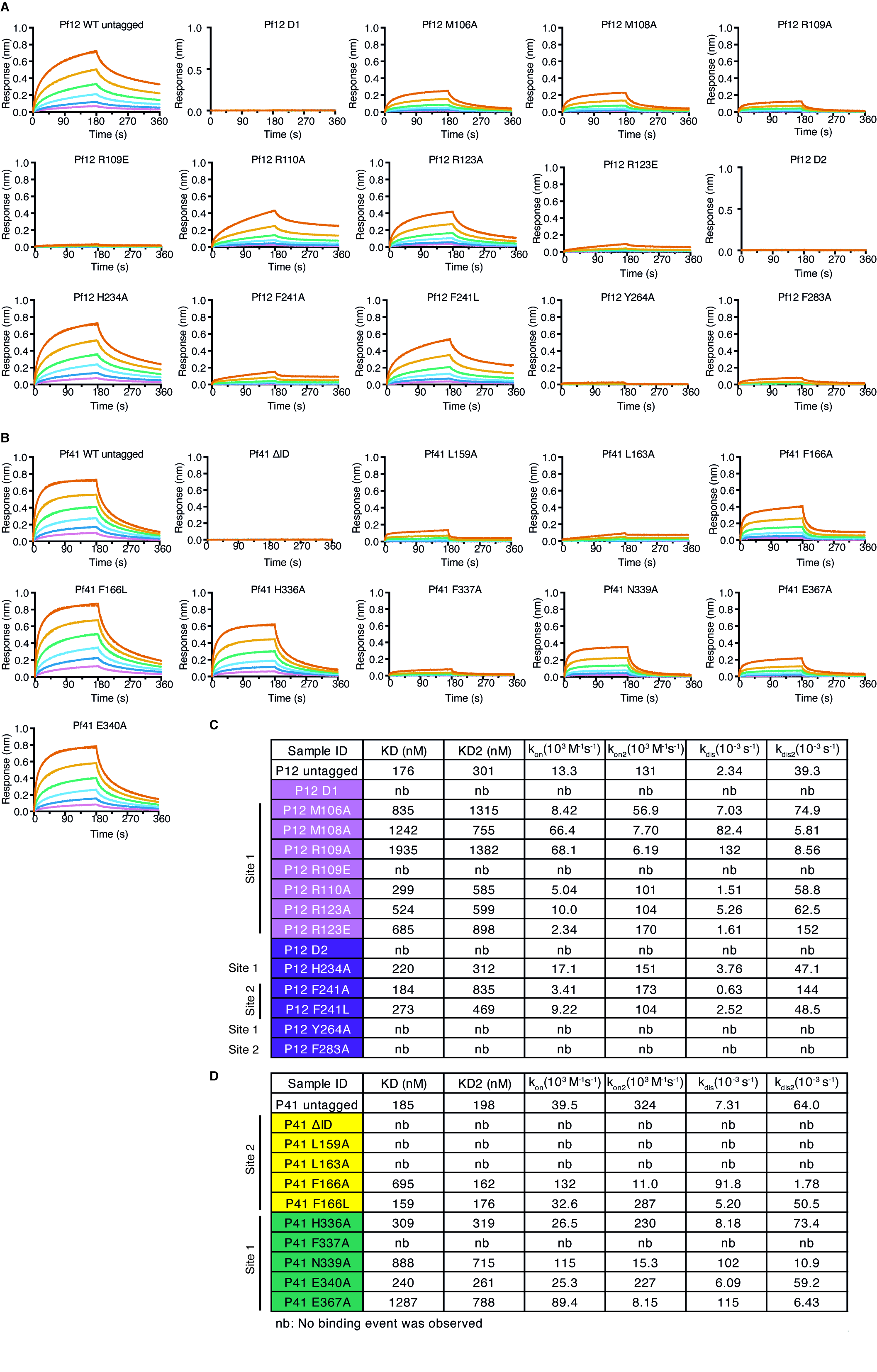

Supplement: xtac005_Supplemental_Files [file xtac005_supplemental_files.zip › S4Fig.tif]

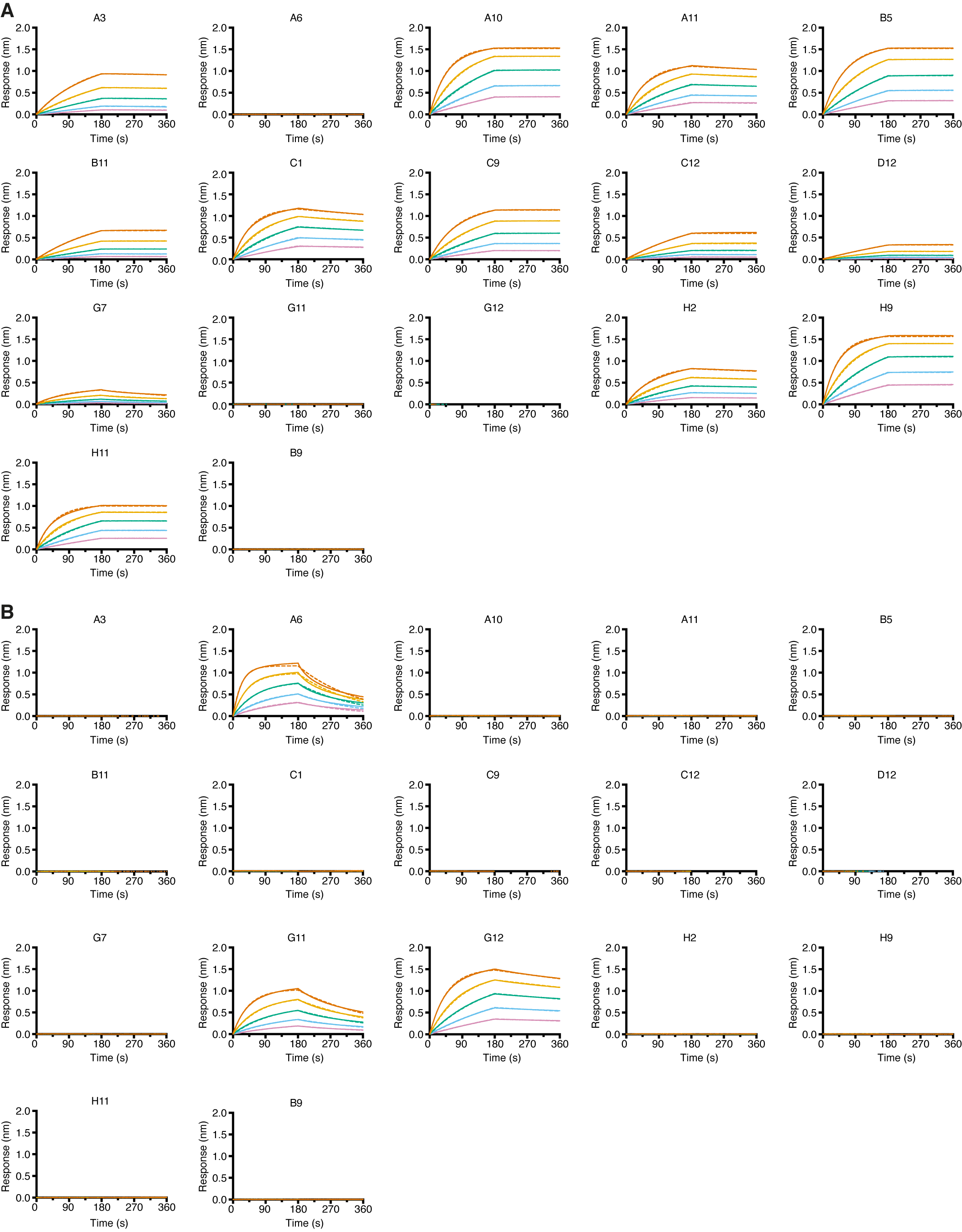

Supplement: xtac005_Supplemental_Files [file xtac005_supplemental_files.zip › S5Fig.tif]

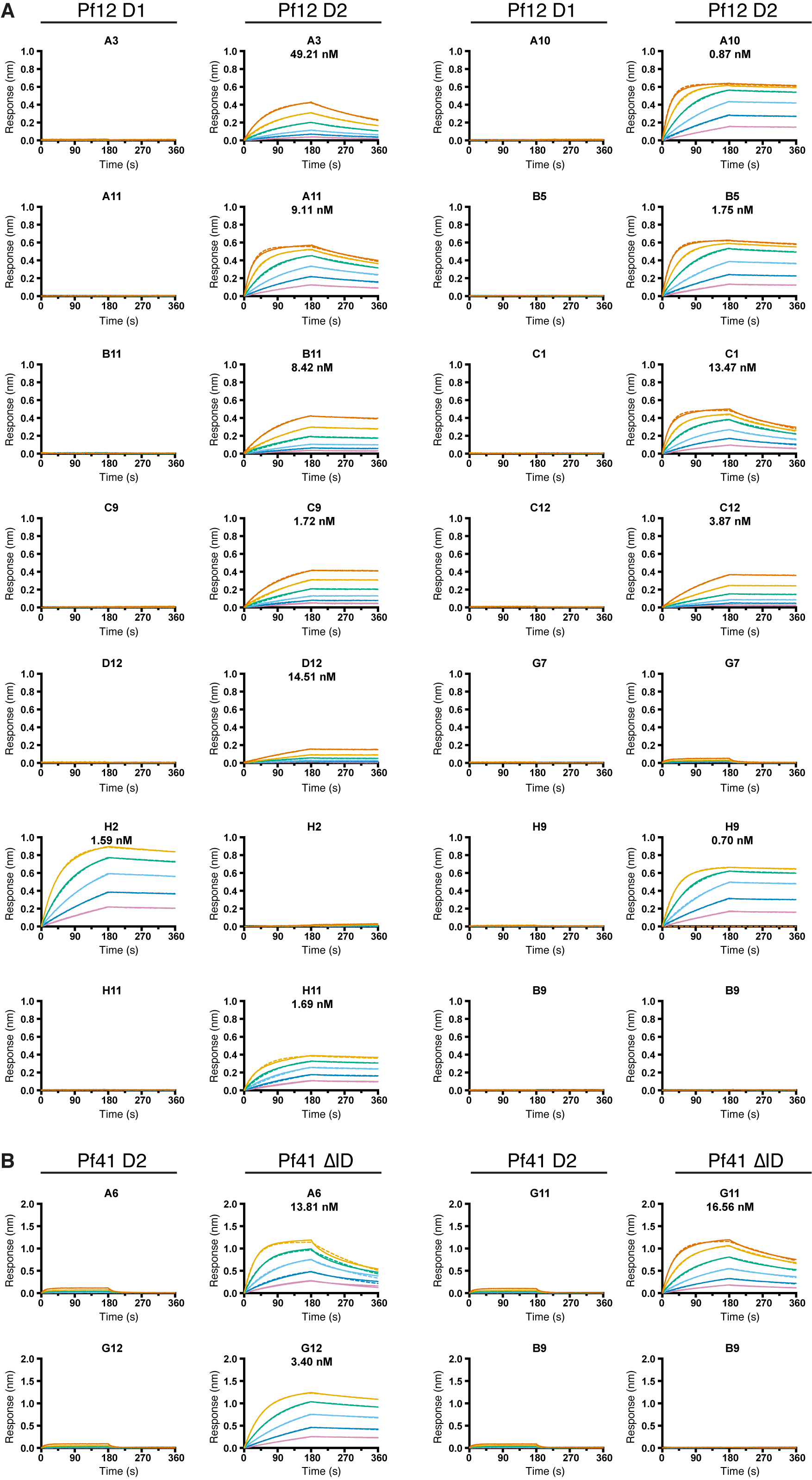

Supplement: xtac005_Supplemental_Files [file xtac005_supplemental_files.zip › S6Fig.tif]

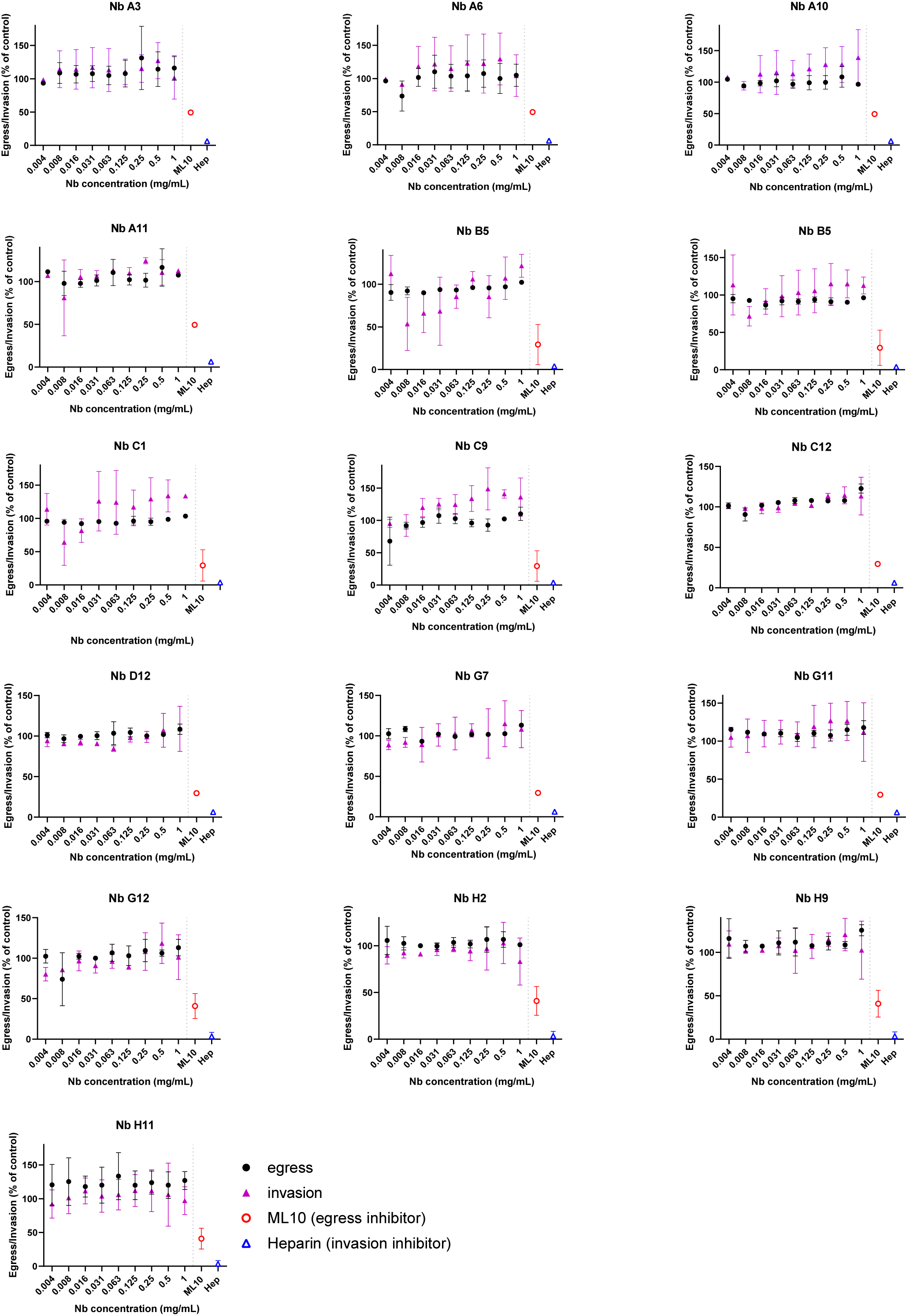

Supplement: xtac005_Supplemental_Files [file xtac005_supplemental_files.zip › S7Fig.tif]
